# Modeling Myeloid Malignancies Using Zebrafish

**DOI:** 10.3389/fonc.2017.00297

**Published:** 2017-12-04

**Authors:** Kathryn S. Potts, Teresa V. Bowman

**Affiliations:** ^1^Department of Developmental and Molecular Biology, Albert Einstein College of Medicine, Bronx, NY, United States; ^2^Gottesman Institute for Stem Cell Biology and Regenerative Medicine, Albert Einstein College of Medicine, Bronx, NY, United States; ^3^Department of Medicine (Oncology), Albert Einstein College of Medicine, Bronx, NY, United States

**Keywords:** splicing, myelodysplastic syndrome, acute myeloid leukemia, zebrafish, hematopoiesis, malignancies

## Abstract

Human myeloid malignancies represent a substantial disease burden to individuals, with significant morbidity and death. The genetic underpinnings of disease formation and progression remain incompletely understood. Large-scale human population studies have identified a high frequency of potential driver mutations in spliceosomal and epigenetic regulators that contribute to malignancies, such as myelodysplastic syndromes (MDS) and leukemias. The high conservation of cell types and genes between humans and model organisms permits the investigation of the underlying mechanisms of leukemic development and potential therapeutic testing in genetically pliable pre-clinical systems. Due to the many technical advantages, such as large-scale screening, lineage-tracing studies, tumor transplantation, and high-throughput drug screening approaches, zebrafish is emerging as a model system for myeloid malignancies. In this review, we discuss recent advances in MDS and leukemia using the zebrafish model.

## Introduction

Myeloid malignancies are clonal disorders of hematopoietic stem and progenitor cells in which there is bone marrow failure, an overgrowth of blasts, differentiation arrest, and lineage skewing. These malignancies include chronic disorders such as myelodysplastic syndrome (MDS), myeloproliferative neoplasm (MPN), and chronic myeloid leukemia (CML) and acute conditions such as acute myeloid leukemia (AML). These disorders are distinguished by the prevailing cell type, pathogenic severity, prognosis, and molecular underpinning. The etiology of most myeloid malignancies is poorly characterized; however, recent large-scale sequencing of patient samples has uncovered key recurrent classes of mutated factors (1–4). Through these studies, researchers identified genetic alterations in factors involved with gene expression regulation including hematopoietic transcription factors, spliceosomal components, and epigenetic regulators. Although genotype–phenotype correlations between a mutated gene and disease state are highly suggestive of causation, model organisms provide a controlled approach to demonstrate the connections between genetic alteration and blood defects as well as to determine the underlying mechanism in more uniform genetic backgrounds. Since the establishment of the first model of transplantable *c-myc*-driven T-cell acute lymphoblastic leukemia (T-ALL) (5), the zebrafish *Danio rerio* has emerged as a useful animal model to explore the control of both normal and malignant hematopoiesis.

## Zebrafish Hematopoiesis

Most of the core regulators of hematopoiesis are evolutionarily conserved between teleosts, such as zebrafish, and mammals, such that findings in zebrafish can be directly translated into mouse and human systems. According to the recently completed and updated annotation of the zebrafish genome, approximately 70% of protein-coding genes in humans have at least one ortholog in the zebrafish, and 84% of disease-associated genes have a zebrafish equivalent (6). This extent of homology further demonstrates the potential utility of zebrafish to define critical regulators of malignancies and the underlying genetic causes. Having an in-depth understanding of the normal processes and signaling requirements underpinning hematopoietic lineage emergence and development provides a solid framework to understand how genetic perturbations exert their influence in disease states. Studying hematopoiesis during embryonic development can be advantageous to minimize the accumulation of environmental influences acquired through the life of an organism. Utilizing the zebrafish model to study embryonic hematopoiesis has a myriad of advantages including high fecundity, rapid external embryonic development, organismal transparency, numerous hematovascular fluorescent reporter lines, genetic tractability, and similar chronological and spatial lineage emergence kinetics and regulation to mammals.

Like other vertebrates, zebrafish hematopoiesis develops in three discrete waves: primitive, erythro-myeloid progenitor (EMP)-derived, and definitive (7). All three waves of hematopoiesis arise from lateral mesoderm-derived cells that possess different hematopoietic differentiation capacity. The primitive hematopoietic wave arises during the first 24 h post fertilization (hpf) from two locations: the anterior lateral mesoderm generates myeloid lineages, and the intermediate cell mass generates primitive myeloid and erythroid cells. Emergent primitive myeloid cells then migrate around the yolk sac and differentiate into distinct lineages, up-regulating expression of *spi/pu.1, colony stimulating factor 1 receptor* (*csf1r/fms), csf3r, l-plastin*, and *myeloperoxidase* (*mpo/mpx*), while the primitive *gata1*-expressing erythroid cells upregulate the levels of *erythropoietin receptor* (*epor)* and *globin* genes then enter circulation (8–14). The transient EMP wave derives from the posterior blood island and differentiates to form definitive erythroid and myeloid cells that lack self-renewal or multilineage differentiation capacity (15, 16). Despite the mostly transient nature of these waves, new findings from the past several years indicate that some myeloid progenitors from the primitive and EMP wave could persist in adulthood and provide the pool for microglia (macrophages in the brain) and other tissue-resident macrophages (17–19). It will be interesting to see if these embryonically derived cells play a role in human disease.

The final wave of hematopoietic specification gives rise to definitive adult-like hematopoietic stem cells (HSCs), which possess both self-renewal capacity and erythroid, myeloid, and lymphoid potential. Starting from approximately 30 hpf, HSCs emerge from *kinase insert domain receptor-like (kdrl)-*positive endothelium lining the ventral wall of the dorsal aorta, equivalent to the mammalian aorta-gonad-mesonephros region (16, 20, 21). The newly emergent HSCs transiently co-express endothelial markers, such as *kdrl*, and HSC markers, such as *cd41* and the transcription factors *cmyb* and *runt-related transcription factor 1* (*runx1*) (15, 16). From approximately 48–72 hpf, HSCs then migrate *via* the circulation to the caudal hematopoietic tissue (CHT) (22), the functional equivalent of the mammalian fetal liver. Between 48–96 hpf, cells from the CHTs will then seed the thymus for T-cell production or the kidney marrow, which functions as the adult hematopoietic niche similar to the mammalian bone marrow (15).

The zebrafish model affords many advantages for investigating mechanisms underlying normal and malignant myelopoiesis [reviewed in Ref. (23)]. The fate determination, differentiation, and maturation of myeloid cells are highly similar from embryonic development to adulthood. As such, studies of myeloid development in zebrafish have been informative not only for understanding embryonic development but also for adult myeloid malignancies [reviewed in Ref. (23)]. Myeloid precursors express conserved transcription factors such as *spi1/pu.1, runx1*, and *ccaat-enhancer binding protein alpha* (*cebp*α) that are critical in myeloid lineage commitment (11, 24, 25). Mature myeloid cell types with similarities or equivalence to well-defined mammalian lineages have been identified in zebrafish development based on their expression of signature genes, histochemical staining properties, and morphology: macrophages that express genes such as *l-plastin, lysozyme (lyz), and csfr1/fms*; neutrophils that express *mpx and matrix metalloproteinase 9 (mmp9*); and basophils/eosinophils that have high levels of *gata2* expression (9, 24, 26, 27).

Zebrafish also possess many technical advantages. Targeted genetic manipulations allow for rapid alteration of gene function, including anti-sense morpholino knockdown (MO), zinc finger nucleases, transcription activator-like effector nucleases (TALENs), and clustered regularly interspaced short palindromic repeat (CRISPR)/CRISPR-associated protein 9 (Cas9) technologies for precision genome editing [reviewed in Ref. (28)]. Exogenous expression of proteins of interest is also possible in zebrafish, either through transient introduction of *in vitro* transcribed mRNA or through stable integration of DNA, most commonly *via* the Tol2 transposon-based transgenesis system (29, 30). Phenotype-driven genetic or chemical screening approaches are commonly employed due to the large clutch size, rapid generation time, and ease of drug treatment by infusion in the water. External and transparent embryonic development in combination with the multitude of fluorescent reporter lines enables sophisticated *in vivo* live imaging of lineage emergence and cell dissemination. Transparent mutants, such as the *casper* line (31), improve imaging capacity in adult zebrafish. Transplantation of zebrafish-derived hematopoietic tumor cells has been utilized to quantify and define subsets of leukemic propagating cells, as well as to image tumor microenvironmental interactions (32–34).

We can therefore take advantage of the technical and genetic advantages of the zebrafish model to study the genetic basis of malignancy and translate the findings to inform a better understanding of human cancer biology for therapeutic application. Current pathways to develop therapeutics for disease treatment are costly, labor and animal intensive, and take 10–30 years from discovery of the molecule or pathway to having a drug in the clinic. It is therefore essential to utilize streamlined processes for *in vivo* testing of drug targets. The zebrafish model, with high fecundity, conservation of many key genes, and an extensive experimental toolbox, provides a high-throughput model for such *in vivo* analysis.

## Myeloid Malignancies

Myelodysplastic syndrome and AML are among the most common myeloid malignancy of the elderly each affecting 3–5 out of 100,000 people in the USA with approximately 10,000–20,000 newly diagnosed cases per annum (35–39). Both malignancies stem from clonal HSC disorders and are characterized by bone marrow failure and peripheral blood cytopenias. A major distinguishing characteristic of AML is the presence of excessive (>20%) undifferentiated myeloid blast in the bone marrow or peripheral blood, which are generally low in MDS patients. MDS is thought to be a precursor syndrome to AML with up to 30% of MDS cases progressing to secondary AML.

## Zebrafish Models of AML

The classic model of AML development states that cells accumulate molecular alterations (large chromosomal rearrangements or genetic point mutations) in two classes: those that promote proliferation (class I) and those that impair differentiation (class II) (40). Prognostic risk is stratified based on the cytogenetic and molecular mutation profile present in the leukemia. For example, cytologically normal *FMS-like tyrosine kinase 3* (*FLT3*)-*internal tandem duplication* (*FLT3-ITD*) correlates with an adverse prognosis, while *nucleophosmin 1* (*NPM1*) mutations are linked with favorable outcomes. Investigating the molecular mechanisms driving AML is difficult in human samples, thus disease models are examined in model organisms including mouse and zebrafish. The first cancer model established in zebrafish in the early 2000s was *c-myc*-driven T-ALL (5), but since then robust myeloid leukemia models have finally been established. Most of these models are based on exogenous expression of prominent human AML fusion oncogenes derived from chromosomal translocations. These oncogenes are generally considered to be potent drivers of AML, with expression of such mutations in zebrafish often resulting in severe, early-arising, embryonic lethal hematologic anomalies, which preclude the study of adult leukemia. Despite this limitation, by employing the pliable genetic and chemical advantages of studies in embryonic zebrafish, much has been discovered regarding underlying mechanisms of these AML-like phenotypes.

## Chromosomal Rearrangements in Zebrafish AML Models

**Figure 1:**
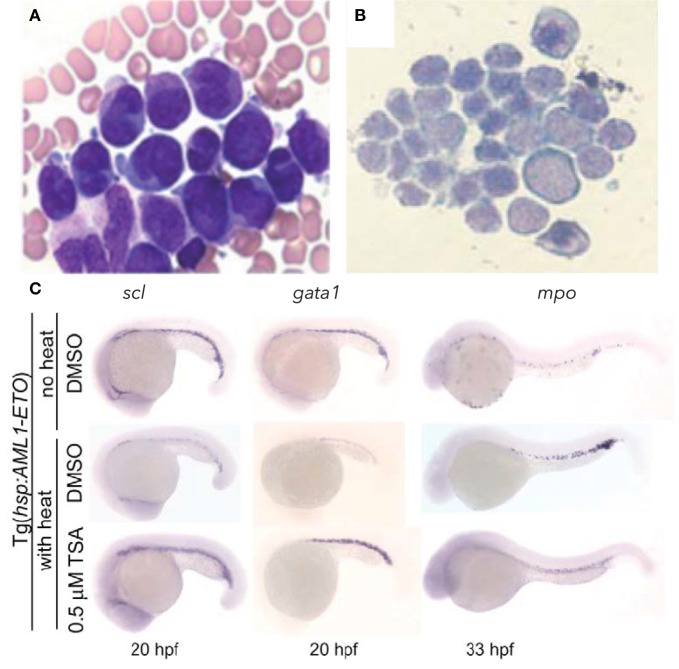
Hematopoietic phenotype conserved in zebrafish model of AML-ETO driven AML. **(A,B)** Wright–Giemsa stained blood cells from **(A)** human acute myeloid leukemia (AML) patient bone marrow smear demonstrating accumulation of promyelocytes [modified and published with permission from Ref. (52)] and **(B)** zebrafish peripheral blood smear showing accumulation of myeloid blasts from AML-ETO overexpressing embryos at 40 hpf. **(C)** Rescue of hematopoietic phenotype with trichostatin A (TSA) treatment. Inducible Tg[*hps:AML1-ETO*] line utilized, such that heat-shocked Tg embryos treated with DMSO develop AML-like phenotype, which can be reversed with TSA treatment. *scl* marks hematopoietic stem and progenitor cells; *gata1* marks erythroid lineage; *mpo* marks myeloid lineage. Panels **(B,C)** modified and published with permission from Yeh et al. (50).

The chromosomal translocation t(8;21)(q22;q22) was one of the first molecular alterations identified in AML, with a frequency of 5–15% of all human AML cases. It results in the fusion of two nuclear proteins: acute myeloid leukemia 1 protein (AML1, also called RUNX1/CBFα2/PEBPαB) and eight twenty one [ETO, or myeloid translocation gene on chromosome 8 (MTG8/RUNX1T1)]. AML1 is a master transcriptional regulator of definitive hematopoiesis that binds enhancers and activates hematopoietic gene expression (41). Chromosomal translocations and mutations involving *AML1* are associated with several forms of adult leukemia and childhood MDS (42, 43). ETO is broadly expressed in hematopoietic cells, including CD34^+^ progenitors, and acts as a nuclear localized zinc finger containing protein that normally recruits the nuclear receptor co-repressor/SIN3/histone deacetylase (HDAC) complex to induce transcriptional repression, including of *AML1* (44–47). The AML1-ETO molecular subtype of leukemia is characterized by granulocyte precursor accumulation (48, 49). In zebrafish, transient induction of the human *AML1-ETO* oncogene during development could recapitulate the granulocytic lineage skewing observed in human patients (Figures 1A,B) (50). *AML1-ETO* expressing embryos displayed a biased expression of *spi1/pu.1* in myelo-erythroid progenitors at the expense of *gata1*, resulting in an expansion of granulocytes (Figure 1C). This largely recapitulated both the differentiation changes observed in human patients and the phenotype in the mouse model (51), demonstrating the utility of the zebrafish system. Mechanistic studies revealed that the observed lineage skewing was mediated *via* modulation of the early fate choice transcription factor *stem cell leukemia (scl)*. Yeh and colleagues then took advantage of the screening capability of the zebrafish and performed an unbiased chemical suppressor screen to find small molecules that could reverse the myeloid expansion in *AML1-ETO*-expressing zebrafish. They identified that cyclooxygenase 2 (COX-2) and β-catenin pathways were downstream of AML1-ETO and that HDAC inhibition by trichostatin A could therapeutically target the AML-like effects in the zebrafish model (Figure 1C).

A zebrafish model of the chromosomal translocation t(9;12)(p24;p13) fusion oncogene ETS leukemia virus 6 (ETV6) and Janus kinase 2 (JAK2) has also been generated. ETV6 (also known as TEL) is an E26 transformation-specific (ETS) family transcription factor involved in early embryonic yolk sac angiogenesis and multilineage adult hematopoiesis including HSC survival (46, 53, 54). JAK2 is a non-receptor tyrosine kinase commonly involved in hematopoietic cytokine signaling cascades and crucial in erythro-myeloid differentiation and HSC maintenance and function (55). The TEL-JAK2 fusion product leads to constitutive activation of JAK2 kinase activity (56). TEL-JAK2 has been identified in lymphoid and myeloid malignancies, with fusion between TEL exon 5 and JAK2 exon 9 occurring in T-ALL, while TEL exon 5 is found to fuse with JAK2 exon 12 in CML (57). To generate a myeloid-restricted mutant zebrafish line, Onnebo and colleagues expressed the *tel-jak2a* fusion oncogene under the control of the *spi1/pu.1* promoter (58). These transgenic animals have disrupted embryonic hematopoiesis, including anemia and expansion of the myeloid compartment. Of note, a subsequent study found that expression of the TEL exon 5-JAK2 exon 9 variant led to lymphoid-restricted defects, while expression of the TEL exon 5-JAK2 exon 12 variant produced myeloid-restricted phenotypes, consistent with prior clinical observations (59). These results indicate that the lineage selection for the specific TEL-JAK2 variant occurs *via* regulation of the downstream signaling rather than at the level of the chromosomal aberration.

## Non-Fusion Oncogenes in Zebrafish AML Models

Gain-of-function mutations in *FLT3* occur in ~30% of AML cases and correlate with poor prognosis (60). Mutations include the internal tandem duplication (FLT3-ITD) and point mutations in the tyrosine kinase domain (FLT3-TKD), both of which result in elevated tyrosine kinase activity (61, 62). FLT3 (also known as FLK2 and STK1) is expressed in human HSCs and is essential for adult HSC and immune hemostasis (63). He et al. established the function of zebrafish *flt3* in hematopoietic development, demonstrating that MO knockdown of endogenous *flt3* in zebrafish significantly impaired progenitor and myeloid differentiation (52). Transient expression of human *FLT3-ITD via* mRNA injections into embryos resulted in expansion of myeloid progenitors (*pu.1*^+^) and mature cells (*mpx*^+^ and *cebp*α^+^). Elevation of downstream signaling such as phosphorylation of Stat5, Erk1/2, and Akt was also observed indicating human FLT3-ITD can trigger established endogenous signals of Flt3 in the zebrafish. Transient expression of human FLT3-TKD (D835Y) also resulted in myeloid cell expansion, but to a lesser extent than the FLT3-ITD. To demonstrate the ability of the zebrafish to test relevant human drugs, He et al. treated FLT3-ITD and FLT3-TKD expressing zebrafish embryos with AC220, a tyrosine kinase inhibitor shown to have potent selectivity for FLT3 (52, 64). Consistent with inhibiting the kinase domain of FLT3, they found that AC220 partially rescued the myeloid effects of FLT3-ITD; however, this did not abrogate the FLT3-TKD phenotype. Subsequently, Lu and colleagues generated a stable transgenic line with myeloid-restricted (*spi1/pu.1* promoter*-*driven) FLT3-ITD and found that these animals develop adult AML symptoms, further illustrating the conservation of function of this oncogene from zebrafish to humans (65).

Modeling of non-fusion oncogenes is also underway in zebrafish. NPM1 is a ubiquitously expressed nucleolar phosphoprotein that regulates multiple cellular processes and is the most frequently mutated gene in adult AML, occurring in ~30% of cases (4, 66). Mutations in *NPM1* result in altered protein localization from the nucleus to the cytoplasm (termed *NPMc*^+^). Zebrafish have two *NPM1* orthologs, *npm1a* and *npm1b* (67). Double MO knockdown of both paralogs results in the production of fewer myeloid cells. Global transient expression of human *NPMc*^+^, the human mutant cytoplasmic protein, but not wild-type *NPM1*, resulted in increased *spi1/pu.1*^+^ myeloid precursors, *mpx*^+^ granulocytes and *csf1r*^+^ macrophages (67). Of note, the myeloid expansion from *NPMc*^+^ expression was enhanced in a *p53*-deficient background, suggesting that too much *NPMc*^+^ could trigger apoptosis. In line with this finding, a recent study showed that NPM1 acts as a scaffold for the apoptotic apparatus termed the PIDDosome [p53-induced death domain-containing protein 1—receptor-interacting protein-associated ICH-1/CED-3 homologous protein with a death domain (PIDD-RAIDD)-caspase-2 complex] (68). *NPMc*^+^ expression also increased HSC levels within the dorsal aorta, indicating a possible role for mutated NPM1 in leukemic stem cell development. Additionally, *NPMc*^+^ was shown to activate canonical Wnt signaling in early zebrafish development, which contributed to hematopoietic cell expansion (69). The elevation of WNT signaling was confirmed in human *NPMc*^+^ AML blasts, which was reversed by knockdown of the mutant *NPMc*^+^ transcript. Together these finding illustrate how studies in zebrafish embryogenesis can inform mechanism in human AML.

Mutations in *isocitrate dehydrogenase 1 and 2* (*IDH1/2*) are found in ~8% of AML cases (70). IDH1/2 are enzymes that catalyze the oxidative decarboxylation of isocitrate producing α-ketoglutarate. AML-associated mutations in *IDH1/2* perturb this function, altering the citric acid cycle, and leading to production of the oncometabolite 2-hydroxyglutarate, which alters DNA methylation *via* inhibition of ten-eleven translocation 2 (TET2) (70, 71). When *idh1* levels were diminished in zebrafish using morpholino or TALEN approaches, Shi et al. observed expansion of *pu.1*^+^ precursors, impaired myeloid differentiation and reduced HSC formation (72). In contrast, when *idh2* was diminished, the zebrafish displayed similar myeloid cell defects to *idh1* mutants, but normal formation of HSCs, indicating a functional redundancy between the two *idh* factors during early embryonic HSC formation. Expression of human oncogenic IDH1-R132H in wild-type zebrafish induced myeloid compartment expansion that was suppressed by treatment with the potent and selective IDH inhibitor AGI-5198. These studies demonstrate that leukemogenic pathways are conserved between humans and zebrafish and illustrate how zebrafish can be used for therapeutically relevant drug studies.

## Adult AML Models in Zebrafish

These embryonic models demonstrate that partial AML phenotypes can be recapitulated in embryonic zebrafish, which can be useful for mechanistic studies and drug discovery, but do not represent a full adult-arising leukemia. The first adult model of AML in zebrafish was based on the inv(8)(p11;q13) chromosomal translocation resulting in the oncogenic fusion of MYST3 (also known as MOZ, YBFR2, SAS2, TIP60 family histone acetyltransferase monocytic leukemia 3) and nuclear co-activator 2 [NCOA2, also called transcriptional mediator/intermediary factor 2 (TIF2)]. MYST3 is in the MYST family of histone acetyltransferases, while NCOA2 is a member of the p160 HAT family [reviewed in Ref. (73)]. To promote AML formation in zebrafish, Zhuravleva and colleagues expressed the human MYST3-NCOA2 (referred to as MOZ-TIF2) oncogene under the zebrafish *spi1/pu.1* promoter (74). This resulted in development of AML after 14–26 months with immature myeloid blast accumulation in the kidney marrow, but decreased progenitors and lymphocytes in the spleen. However, such an AML phenotype was a rare event (2/180), indicating inefficient transformation, insufficient expression levels driven from the *pu.1* promoter, or perhaps the requirement for a secondary mutation for disease development. Due to the long latency and low penetrance, there have not been further studies with this model.

The t(7;11)(p15;15) chromosomal translocation leading to *nuclear pore complex protein 98* (*NUP98*)*-homeobox protein A9* (*HOXA9*) oncogenic fusion is widely observed in hematological pathologies including MDS, CML, and AML, and correlates with poor prognosis. NUP98 is involved in nuclear trafficking (75), and HOXA9 is a vertebrate transcription factor essential in hematopoiesis with >80% of human AML showing overexpression of *HOXA9* (76). Utilizing a novel Cre-LoxP system that allowed for both myeloid-restricted and heat-shock inducible expression [T*g*(*spi1:loxP-EGFP-loxP:NUP98-HOXA9*); *Tg(hsp70:cre)*], Forrester and colleagues were able to explore the effects of conditional expression of the human *NUP98-HOXA9* both in the embryo as in the studies described above, but also in adulthood. The later inducible expression is key to circumvent any deleterious events from early embryonic expression that could preclude analysis of disease in older animals. Similar to the mouse model (77), induction of human *NUP98-HOXA9* expression resulted in ~23% of transgenic fish developing preleukemic MPN by 2 years (78). Examination of embryos with *NUP98-HOXA9* expression revealed early lineage skewing where *pu.1*^+^ myeloid progenitors were enhanced at the expense of *gata1*^+^ erythroid progenitors, with perturbed myeloid lineage differentiation. A follow-up study that employed chemical approaches to determine the driving mechanism as well as potential new therapeutics for AML revealed that *NUP98-HOXA9* upregulates *prostaglandin synthase 2* (*ptgs2*) to expand HSC numbers (79), a pathway identified in prior studies to be important for normal HSC formation (80, 81). Blocking prostaglandin production with COX inhibitors could reverse the increase in HSCs, suggesting a role for this pathway in leukemic stem cell expansion. Additionally, gene expression analyses showed *NUP98-HOXA9* elevated the expression of the epigenetic modifier *dna methyltransferase 1 (dnmt1)*, which lead to hypermethylation. Treatment with an HDAC inhibitor reversed this phenotype. Both of these pathways were also identified as suppressors of myeloid expansion in the AML-ETO model, suggesting that they could play a more general role in AML induction.

## Modeling MDS in Zebrafish

Myelodysplastic syndromes are a diverse group of chronic myeloid pathologies defined by perturbed clonal hematopoiesis, impaired differentiation and peripheral blood cytopenias with the potential to transform into AML. Substantial research efforts have been invested in understanding the drivers of MDS toward improving diagnosis and stratification of subtypes, which will improve treatment of patients. Until recently the etiology of the heterogeneous clinical outcomes of MDS was unclear. Extensive genomic analyses in recent years have revealed that some subtypes of MDS correlate strongly with mutations in spliceosomal or epigenetic factors (1–3, 82). Mutations in spliceosomal machinery are common and thought to be critical drivers in MDS pathogenesis. They have been identified in approximately 60% of all MDS cases (2), with mutations in splicing factor 3B, subunit 1 (SF3B1) observed in 80–90% of cases with the refractory anemia with ringed sideroblast (RARS) subtype (1, 3, 83, 84). Epigenetic factors are mutated in approximately 45% of MDS cases (2) with mutations in the methylcytosine dioxygenase TET2 being the most prevalently observed in 30% of MDS (2, 82). The function of splicing and epigenetic factors in MDS is still elusive as their role in normal hematopoietic development is unclear. The best way to clarify their function is to generate and study *in vivo* animal models to gain an organism-wide context of normal and perturbed gene function throughout lineage emergence, differentiation, and niche interactions.

## Spliceosomal Components in MDS

**Figure 2:**
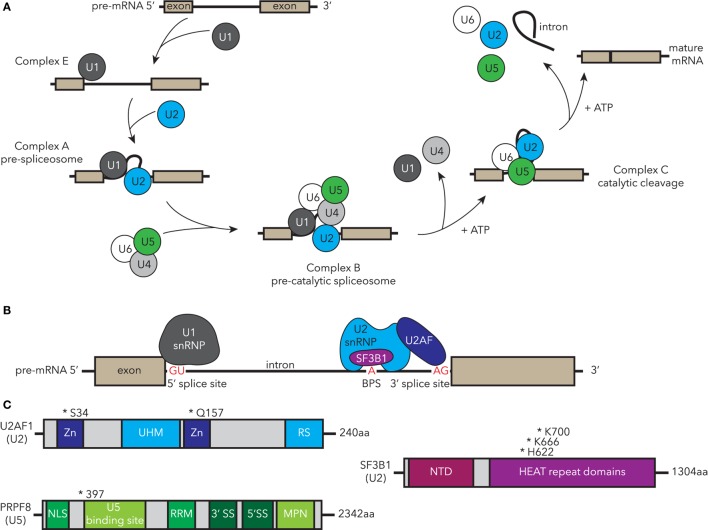
Human MDS-associated mutations in essential components of the spliceosome are conserved in zebrafish disease models. **(A)** Spliceosomal processing of pre-mRNA to mature transcript, indicating recruitment of snRNP complexes. Complexes containing MDS-mutated factors that have been studied in zebrafish are highlighted in blue (U2) and green (U5). **(B)** Essential components of complex A, including binding of the U1 snRNP to the 5′ splice site (SS), and U2 snRNP U2AF recognition and binding of AG in the 3′ SS, while SF3B1 recognizes the branch point site (BPS). **(C)** Structure and common MDS-associated mutations in U2-associated components U2AF1 and SF3B1 and U5 PRPF8. Zn, zinc finger domain; UHM, U2AF homology motif; RS, arginine-serine domain; NTD, N-terminal domain; NLS, nuclear localization sequence; RRM, RNA recognition motif; MDS, myelodysplastic syndrome; snRNP, small nuclear ribonucleo-protein; U2AF1, U2 small auxiliary factor 1; SF3B1, splicing factor 3B, subunit 1; PRPF8, pre-mRNA processing factor 8.

The spliceosome is a large complex within eukaryotic nuclei encompassing five small nuclear ribonucleo-proteins (snRNPs) comprising RNAs and the associated protein molecules [reviewed in Ref. (85)]. The components, structure, and the function of the spliceosome are highly conserved throughout evolution in yeast, teleosts, and mammals. The high conservation of the spliceosome permits experiments from across eukarya to inform human spliceosome function and regulation. The function of the spliceosome is to remove introns from newly transcribed pre-mRNAs, resulting in mature mRNAs that are then translated by ribosomes to generate proteins. Splicing is a dynamic, highly coordinated process, thus its correct action is essential for normal functioning of cells. The major U2-type spliceosome comprises U1, U2, U4, U5, and U6 snRNPs (Figure 2A) and catalyzes the majority of splicing events, while the U12-type minor spliceosome has a specific target subset. Alternative splicing to generate multiple transcript variants for each gene occurs normally throughout development and is regulated in a tissue-specific manner. However, it can also occur as a result of spliceosomal dysfunction. MDS-associated mutations in spliceosomal components can lead to specific alternative splicing events, which correlate with their function in splicing. For example, SF3B1-containing complexes bind the branch point site within introns, and cells with MDS-associated SF3B1 mutations show defects in branch site selection, which can result in alternative proteins or unstable mRNA (Figure 2B) (86–90).

Splicing factor 3B, subunit 1 is a core component of the U2 snRNP and is one of the most highly mutated spliceosomal factors in MDS (2, 82). In addition to MDS, mutations in *SF3B1* have been identified in other types of leukemia such as CLL (91, 92), and several solid organ malignancies, including pancreatic cancer (93), breast cancer (94, 95), and uveal melanoma (96, 97). Mutations in *SF3B1* are strongly correlated with the ring sideroblast phenotype in MDS and are associated with better prognostic outcomes including a decreased risk of AML evolution (82, 83, 98). In SF3B1, most mutations cluster within the HUNTINGTON-ELONGATION FACTOR 3-PR65/A-TOR (HEAT) repeats in the C-terminus of the protein particularly in residues K700, K666, and H662 (Figure 2C) (83, 99). Recent data suggest that the HEAT repeat domains mediate protein–protein interactions (90). How these point mutations alter SF3B1 function and why this leads to hematologic dysfunction is unclear in part due to the limited understanding of the general signaling mechanism through which SF3B1 usually regulates hematopoiesis. To address this latter question, an *sf3b1* loss-of-function zebrafish mutant was studied to understand the normal function of Sf3b1 in hematopoiesis and development (100). The homozygous *sf3b1^hi3394a^* loss-of-function mutants displayed an arrest of primitive hematopoiesis in both myeloid and erythroid lineages, which occurred after specification presenting as a block in differentiation and proliferation. In contrast, specification of definitive HSCs was hindered, despite the normal specification and differentiation of the non-hemogenic endothelial cells within the dorsal aorta. The lower production of mature blood cells coupled with poor HSC output was reminiscent of an MDS phenotype. HSC emergence from hemogenic endothelium is a NOTCH-dependent process (101). NOTCH signaling was normal in *sf3b1* mutant zebrafish, indicating that the HSC induction defect is downstream or NOTCH-independent. These studies establish the importance of Sf3b1 somewhat selectively in hematopoiesis as other tissues such as the vasculature develop normally. How MDS-associated point mutants behave in this context requires further study. Recently, murine models of the most common MDS-associated point mutation (*Sf3b1^+/K700E^*) were generated and can be used to follow-up potential mechanisms identified in unbiased screening systems such as the zebrafish or human cell culture (102, 103).

U2 small auxiliary factor 1 (U2AF1) is mutated in 8–20% of MDS patients with the most common mutations occurring at residues S34 and Q157 (Figures 2B,C) (2, 99, 104, 105). Mutated U2AF1 in MDS causes aberrant splicing and is associated with increased risk of AML evolution. During splicing, SF3B1 interacts with the U2AF complex to help establish the 3′ splice site and splicing fidelity (Figure 2B) (106). Similar to *sf3b1* mutants, homozygous loss-of-function *u2af1^hi199^* mutant zebrafish have fewer definitive HSCs, develop anemia, and have elevated *tp53* transcript levels, phenotypes which are all observed in MDS (107). Knockdown of *tp53 via* MO injections suppressed these hematologic defects suggesting it as a downstream mediator of *u2af1* phenotypes. This model can therefore be used to further dissect the mechanism underlying U2AF1 and p53 activation in MDS.

Recurrent point mutations in the pre-mRNA processing factor 8 (PRPF8) have been reported in MDS and AML, correlating with increased myeloid progenitors, ring sideroblasts, and overall poor prognosis (108, 109). PRPF8 is a highly conserved component of the U5 snRNP that plays a role in both U2- and U12-spliceosomal processing (Figures 2A,C) (110). A zebrafish loss-of-function *prpf8* mutant (called *cephalophonus/cph^gl1^*) was identified through a forward genetic screen for factors that regulate embryonic myelopoiesis (111). The *cph/prpf8* homozygous mutants have defective myeloid and erythroid development, but unlike *sf3b1* and *u2af1* mutants, they show normal formation of definitive HSCs.

These spliceosomal mutants have some overlapping, but also distinct phenotypes. These findings are consistent with what is observed clinically; patients harboring mutations in different splicing factors share some disease features, but also have distinguishing characteristics. This suggests that although all factors are part of the spliceosome, their individual functions either within or outside of the spliceosome contribute to specific facets of disease. Using these zebrafish models will permit unbiased mechanistic explorations into these functions.

## TET2 in MDS

Epigenetics is the study of changes in gene expression patterns regulated by non-genomic modifications without altering the DNA sequence. Epigenetic marks are transmitted through DNA methylation, histone modifications including acetylation and methylation of histone tails, RNA interference, and nuclear organization, thereby modulating transcriptional activation and silencing [reviewed in Ref. (112)]. Such epigenetic marks are heritable, allowing for transgenerational inheritance of non-genetic traits. Epigenetics has established critical roles in embryonic development, maternal/paternal gene imprinting, X inactivation, and disease. In cancer, there is a high prevalence of DNA hypermethylation and histone modification [reviewed in Ref. (113)]. Specifically in MDS and AML, many of the top class of mutated factors are epigenetic modifiers, which have opened the hematology field to delve into the role of epigenetics in normal and diseased hematopoiesis. The factors controlling epigenetic patterns and inheritance are highly conserved between teleosts and mammals, making zebrafish an excellent model to explore how mutations in this process lead to hematologic dysfunction.

Ten-eleven translocation proteins (TET1/2/3), a family of methylcytosine oxidases, function as epigenetic regulators of the genome methylation state. They catalyze the oxidation of 5-methylcytosine to 5-hydroxymethylcytosine, 5-formylcytosine, and 5-carboxycytosine (114), which are key intermediates in DNA demethylation. Controlled methylation and de-methylation are crucial for embryonic development and control of gene expression (115). Somatic deletions and loss-of-function mutations in TET2 frequently occur in myeloid malignancies: ~30% of MDS and ~10% of *de novo* AML cases. *Tet2*-deficient mouse models have shown the function of TET2 in HSC self-renewal and differentiation, with myeloid defects reminiscent of MDS and AML (116, 117). Zinc finger nuclease technology was utilized to generate a homozygous *tet2* loss-of-function zebrafish (118). Consistent with *Tet2* null mice, *tet2*-deficient zebrafish are viable and have intact embryonic hematopoiesis. Similar to murine models and humans, the *tet2*-mutant zebrafish develop progressive clonal myelodysplasia, anemia, and myeloid progenitor expansion as they age. By 24 months, they develop a more severe MDS phenotype including peripheral blood erythrocyte dysplasia. A study in compound mutants for *tet* family members uncovered a redundancy of *tet2* and *tet3* in HSC formation (119). The underlying mechanism for the diminished levels in *tet2;tet3* double mutants was *via* regulation of NOTCH signaling in aortic endothelial cells (119). In mammalian blood cells, TET2 and TET3 are the predominantly expressed TET family members and might act redundantly (120). These data suggest a high degree of similarity in zebrafish and mammalian TET usage in hematopoiesis. Thus, the zebrafish *tet2* single and *tet2;tet3* double mutants will be useful for screening for new treatment targets of this epigenetic driver of MDS.

## 5q− Syndrome and Ribosomopathies

5q− syndrome is an MDS subtype with macrocytic anemia arising due to large deletions within chromosome 5 [reviewed in Ref. (121)]. The deleted chromosomal segment includes two common deleted regions (CDRs) encompassing many genes expressed by HSCs including hematopoietic cytokines, protein phosphatase 2, ribosomal protein S14 (RPS14), heat shock protein family A member 9B (HSPA9B), and more distally NPM1, but which factors are involved in disease phenotypes was unknown for quite some time (122).

The zebrafish mutant *crimsonless (crs)* presents with MDS-like hematological defects from 33 hpf, including anemia with a block in maturation, increased apoptosis and multilineage (erythroid and myeloid) cytopenia (123). The mutation in *crs* was determined to be a point mutation in the *hspa9b* (*hsp70*) gene likely generating a null allele. Hspa9b is a mitochondrial matrix chaperone whose loss leads to blood-restricted oxidative stress and apoptosis. In humans, the *HSPA9B* gene is located within the 5q31 CDR in human MDS. A recent study in human hematopoietic progenitors showed that similar to the zebrafish mutant depletion of HSPA9B in human hematopoietic progenitors also leads to apoptosis (124). Combined these studies suggest that loss of *HSPA9B* could contribute to 5q− syndrome MDS.

In 2008, Ebert and colleagues identified RPS14 as a major driver of 5q− anemia (125). A zebrafish homozygous *rps14* loss-of-function mutant also develops anemia with a terminal erythroid maturation defect equivalent to that observed in 5q− syndrome (126). The *rps14* mutant displayed elevated p53 activity, which was shown to contribute to the later events of the anemia. Mutations in another ribosomal protein RPS19 are linked with the childhood disease Diamond–Blackfan anemia (DBA) (127, 128). Similar to depletion of *rps14*, zebrafish homozygous *rps19* loss-of-function mutants develop a p53-dependent anemia (129). These models will therefore be useful to dissect the signaling pathway intermediates between ribosomal proteins and p53-mediated factors that drive anemia. Indeed, l-leucine, a drug in testing for DBA (130, 131), has already proven effective in treatment of both mutant Rps14- and Rps19-driven anemia in zebrafish (126, 132).

## Conclusion and Future Directions

The zebrafish model has great utility for investigating driver mutations underlying disease pathogenesis in MDS and AML. In particular, the high frequency of spliceosomal mutations identified in human MDS and AML and the conservation of myelo-erythroid phenotypes in the mutants studied to date indicates that this is a useful system to investigate the role of the spliceosome and epigenetics in myeloid malignancies. The above discusses some of the zebrafish myeloid disease models currently being studied (Table 1), and with the ability to rapidly generate mutant lines utilizing technologies such as CRISPR/Cas9, many more genes can be investigated in a relatively high-throughput manner. Furthermore, mechanistic studies are faster, cheaper, and higher throughput with *in vivo* testing of drug pathways feasible. This will facilitate more robust testing of targets *in vivo* in a whole organism setting. From this, we can identify and test rational, targeted pathways and therapeutics rather than the aggressive, non-specific cytotoxic chemotherapies utilized in current MDS and AML treatment regimes.

**Table 1:** Zebrafish models of human AML and MDS.

Human mutated factors	Correlating human disease	Zebrafish manipulation	Reference
AML-ETO t(8;21)(q22;q22)	AML	Transgenic expression of human AML-ETO fusion	(50, 133)
TEL-JAK2 t(9;12)(p24;p13)	AML	Transgenic expression of human TEL2-JAK2 fusions	(58, 59)
FLT3-ITD, FLT3 TKD	AML	Transgenic expression of human FLT3-ITD or FLT3-TKD	(52, 64, 65)
NPM1c	AML	Knockdown of zebrafish *npm1* homolog; transgenic expression of human NPM1c	(67, 69)
IDH1/2	AML	Knockdown of zebrafish *idh1* and idh2 homologs; transgenic expression of human IDH1 point mutant	(72)
MYST3-NCOA2 inv(8)(p11;q13)	AML	Transgenic expression of human MYST3-NCOA2 fusion under the *spi1/pu.1* promoter	(74)
NUP98-HOXA9 t(7;11)(p15;15)	AML	Transgenic expression of human NUP98-HOXA9 fusion under the *spi1/pu.1* promoter	(78, 79)
SF3B1	MDS	*sf3b1^hi3394a^* mutant	(100)
U2AF1	MDS	*u2af1^hi199^* mutant	(107)
PRPF8	MDS	*prpf8^gl1^/cephalophonus^gl1^* mutant	(111)
TET2/3	MDS	*tet2^zdf20^, tet2^mk17^, and tet3^k18^* mutants	(118, 119)
RPS14	Ribosomopathy (5q− MDS)	*rps14^zf624^* mutant	(126)
RPS19	Ribosomopathy (DBA)	*rps19^zf556^* mutant	(129, 134)
HSPA9B	Ribosomopathy (5q− MDS)	*hspa9b^unspecified^/crimsonless^unspecified^* mutant	(123)

*AML, acute myeloid leukemia; MDS, myelodysplastic syndrome; ETO, eight twenty one; JAK2, Janus kinase 2; FLT3, FMS-like tyrosine kinase 3; ITD, internal tandem duplication; TKD, tyrosine kinase domain; NPM1, nucleophosmin 1; IDH1/2, isocitrate dehydrogenase 1 and 2; NCOA2, nuclear co-activator 2; NUP98, nuclear pore complex protein 98; HOXA9, homeobox protein A9; SF3B1, splicing factor 3B, subunit 1; U2AF1, U2 small auxiliary factor 1; PRPF8, pre-mRNA processing factor 8; TET2/3, Ten-eleven translocation; HSPA9B, heat shock protein family A member 9B; DBA, Diamond–Blackfan anemia*.

Unlike murine models, which often faithfully recapitulate human leukemias, zebrafish myeloid malignancy models frequently fail to develop the full adult disease state as observed in human MDS and AML, which is a limitation in the system to date. To address this, xenograft models using human leukemic cell lines or primary leukemic cells are now being used in zebrafish to investigate human disease progression (135). As zebrafish younger than a week have an innate immune system, but do not yet have a functioning adaptive immune system, xenografts in larvae are particularly useful to examine cancer progression *in vivo* without the need for damaging pre-conditioning regimens to permit human cell engraftment (23, 136). Thus, xenografts in zebrafish provide a new dimension for analysis of disease states and causative mechanisms, and will be extremely useful moving forward to screen human cells for drug susceptibility within an *in vivo* environment.

Currently, ubiquitous loss-of-function mutants are used to investigate the normal function of genes of interest in development. In human myeloid malignancies, mutations in genes often arise somatically and are missense rather than null. Genetic approaches in murine models permit tissue-specific expression of point mutants, which more closely resembles the human condition. With the advent of CRISPR/Cas9 technologies, the next step in zebrafish is to generate specific knock-in models of disease-associated point mutants [reviewed in Ref. (137)] and to induce mutations in a tissue-specific manner (138). These advances will expand our understanding of MDS and AML, including how faithfully the loss-of-function mutants recapitulate the phenotype of point mutants, and for screening of potential treatment molecules. The recent development of clonal lineage tracing capabilities for the blood system in zebrafish (139) opens the door to uncover drivers of the initial clonal events prior to hematologic dysfunction. Additionally, using live animal imaging, the initiation of cancer at the single-cell level was recently demonstrated in zebrafish melanoma (140). Combining these clonal and genetic approaches in myeloid malignancies can help examine the earliest events of disease formation not readily accomplished in other animal models.

## Author Contributions

KP and TB designed and wrote the manuscript. TB gave final approval of the manuscript.

## Conflict of Interest Statement

This work was supported by the Gabrielle’s Angel Foundation, American Cancer Society RSG-129527-DDC, Kimmel Foundation, the EvansMDS Foundation, and the New York State Department of Health Contract C30292GG. The funders had no role in study design, data collection and analysis, decision to publish or preparation of the manuscript. All authors declare no conflict of interest.
